# TAZ maintains telomere length in TNBC cells by mediating Rad51C expression

**DOI:** 10.1186/s13058-021-01466-z

**Published:** 2021-09-06

**Authors:** Lu Yang, Bo Wang, Xinyan Jiao, Can Zhou, Su Chen, Xiaoqian Gao, Wei Sun, Shaoran Song, Juan Li, Jie Liu, Yaochun Wang, Peijun Liu

**Affiliations:** 1 Center for Translational Medicine, The First Affiliated Hospital of Xi’an Jiaotong University, 277 Yanta Western Rd, Xi’an, 710061 Shaanxi Province China; 2 Key Laboratory for Tumor Precision Medicine of Shaanxi Province, The First Affiliated Hospital of Xi’an Jiaotong University, 277 Yanta Western Rd, Xi’an, 710061 Shaanxi Province China; 3 Department of Breast Surgery, The First Affiliated Hospital of Xi’an Jiaotong University, 277 Yanta Western Rd, Xi’an, 710061 Shaanxi Province China; 4 Laboratory of Molecular and Cellular Biology, School of Basic Medical Sciences, Henan University School of Medicine, North Jinming Avenue, Kaifeng, 475004 Henan Province China

**Keywords:** TAZ, Telomere, Rad51C, TERRAs, Shelterin proteins, TNBC

## Abstract

**Background:**

Telomere maintenance is crucial for the unlimited proliferation of cancer cells and essential for the “stemness” of multiple cancer cells. TAZ is more extensively expressed in triple negative breast cancers (TNBC) than in other types of breast cancers, and promotes proliferation, transformation and EMT of cancer cells. It was reported that TAZ renders breast cancer cells with cancer stem cell features. However, whether TAZ regulates telomeres is still unclear. In this study, we explored the roles of TAZ in the regulation of telomere maintenance in TNBC cells.

**Methods:**

siRNA and shRNA was used to generate TAZ-depleted TNBC cell lines. qPCR and Southern analysis of terminal restriction fragments techniques were used to test telomere length. Co-immunoprecipitation, Western blotting, immunofluorescence, Luciferase reporter assay and Chromatin-IP were conducted to investigate the underlying mechanism.

**Results:**

By knocking down the expression of TAZ in TNBC cells, we found, for the first time, that TAZ is essential for the maintenance of telomeres in TNBC cells. Moreover, loss of TAZ causes senescence phenotype of TNBC cells. The observed extremely shortened telomeres in late passages of TAZ knocked down cells correlate with an elevated hTERT expression, reductions of shelterin proteins, and an activated DNA damage response pathway. Our data also showed that depletion of TAZ results in overexpression of TERRAs, which are a group of telomeric repeat‐containing RNAs and regulate telomere length and integrity. Furthermore, we discovered that TAZ maintains telomere length of TNBC cells likely by facilitating the expression of Rad51C, a crucial element of homologous recombination pathway that promotes telomere replication.

**Conclusions:**

This study supports the notion that TAZ is an oncogenic factor in TNBC, and further reveals a novel telomere-related pathway that is employed by TAZ to regulate TNBC.

**Supplementary Information:**

The online version contains supplementary material available at 10.1186/s13058-021-01466-z.

## Background

Breast cancers can be classified by different receptor status. The three most important receptors are estrogen receptor (ER), progesterone receptor (PR), and Her-2. The cancers expressing none of these receptors are named triple negative breast cancer (TNBC). Accounting for ~ 15–20% of all breast cancers, TNBC shows poorer prognosis than other subgroups owning to highly aggressive metastatic behavior and lack of targeted therapies [1]. Previous studies reported that the expression of TAZ (Transcriptional coactivator with PDZ-binding motif) is higher in TNBC than in the other types of breast cancers [2]. TAZ mainly acts as an effector of Hippo pathway to control organ size by regulating cell proliferation and apoptosis [3]. Accumulating studies indicated that TAZ shows an oncogenic function in breast cancers by promoting cell proliferation, transformation and EMT [4]. TAZ also confers breast cancer cells possessing “stemness” properties and plays crucial roles in the regulation of breast cancer stem cell self-renewal, tumor initiation, metastasis and chemoresistance [5, 6].

The regulation of telomere length has been implicated in “stemness” maintaining in eukaryotes. Telomeres are protective complexes containing tandem nucleotide repeats and multiple binding proteins at the end of eukaryotic chromosomes. In each round of cell division, chromosomes are shortened due to the inefficient DNA duplication at the very end of the chromosomes. Telomeres protect the chromosomes against such replication-associated attrition. The length of telomeres is dynamically changed in various physiological or pathological processes. For instance, telomeres have been reported to be shortened during aging or tumorigenesis [7]. It has been reported that telomeres are protected by shelterin proteins, as well as by telomeric repeat-containing RNAs (TERRAs). Shelterin proteins include 6 components, TRF1, TRF2, TIN2, TPP1, POT1 and RAP1, forming a six-subunit containing complex. Shelterin proteins bind with telomere DNA to form T-loop like structures and protect telomeres from being recognized by DNA repair machineries [8]. Human TERRAs are a group of UUAGGG repeats-containing long noncoding RNAs transcribed from either telomere DNA or intrachromosomal telomeric repeats [9]. TERRAs can localize near to telomeres and hybridize with telomeres to form RNA–DNA heteroduplexes. TERRAs have been recognized to serve as a scaffold for recruiting multiple chromatin factors to telomeres, and plays a central role in telomere maintenance [10].

Telomere length limits the number of cell divisions. Maintaining the integrity of telomeres is essential for the unlimited cancer cell proliferation. In addition, extremely shortened telomeres elicit DNA damage responses that trigger cellular senescence [11]. Overwhelming majority of cancer cells reverse telomere attrition by activating hTERT, the catalytic subunit of the telomerase. Telomerase is a reverse transcriptase enzyme that catalyzes the building of nucleotide repeats to the end of the chromosome, resulting in lengthened telomeres. In some cancers, telomere length is maintained by alternative lengthening of telomere (ALT) mechanism which is based on homologous-recombination (HR) instead of by using telomerase [12]. Therefore, cancer cells can be classified as telomerase-positive or ALT-positive. However, recently, coincidence of telomerase activity and ALT activity in the same cancer cells has been reported [13]. Moreover, Shelterin proteins and TERRAs have also been reported to play critical roles in the regulation of telomere length [14, 15].

In this study, we uncovered a previously unappreciated role of TAZ in the regulation of telomere length in TNBC cells. Moreover, we also explored multiple telomere length regulating factors. Our results showed that TAZ-TEAD directly binds to the promoter of Rad51C and causes an elevated transcription of Rad51C. The increased Rad51C further regulates the telomere length and plays an important role in maintaining telomere integrity. In addition, elevated levels of TERRAs in TAZ-deficient cells may also contribute to telomere shortening.

## Methods

### Cell culture and transfection

All cell lines were purchased from the Cell Bank of Chinese Academy of Sciences. MCF7, MDA-MB-453 and MDA-MB-231 breast cancer cells were cultured in High Glucose DMEM (#SH30022.01, Hyclone) supplemented with 10% FBS (#SH30084.03, Hyclone). T47D, HCC1806 and BT549 were cultured in RPMI-1640 medium (#SH30027.01, Hyclone) with 10% FBS. BT549 cells were additionally supplemented with 1 μg/ml insulin (#I9278, sigma). MCF10A was maintained in DMEM F12 medium (#SH30023.01, Hyclone) supplemented with 5% horse serum (#26050088, Invitrogen), 0.5 μg/ml hydrocortisone (#386698, sigma), 10 μg/ml insulin, and 20 ng/ml recombinant human EGF (#100-47, PeproTech). All cells were incubated in 5% CO_2_ at 37 °C.

Two different siRNAs specifically targeting TAZ were designed and synthesized by GenePharma company (Shanghai, China). pcDNA 3.1(+)-TAZ and pcDNA 3.1(-)-TAZ-4SA overexpression plasmids were kindly provided by Dr. Jianmin Zhang from the Department of Cancer Genetics & Genomics, Roswell Park Cancer Institute, Buffalo, USA. Transfections of siRNAs or plasmids by using Lipofectamine 2000 (#11668-019, Invitrogene) were performed according to the manufacturer’s protocol. To generate the TAZ knockdown stable transfectants, TAZ-specific lentiviral shRNAs inserted into the GV112 vector (hU6-MCS-CMV-Puromycin) were designed and constructed by GenePharma company (Shanghai, China), and cells were transfected in the presence of 5 μg/ml polybrene according to the manufacturer’s instruction. The sequences of the siRNA and shRNA oligonucleotides are provided in Additional file 1: Table S1.

### Western blot analysis and antibodies

Cells were lyzed using RIPA buffer (#P0013C, Beyotime, Shanghai, China) supplemented with Protease and Phosphatase Inhibitors (#A32959, Thermo). Proteins were transferred to PVDF membranes after being separated in SDS/PAGE. The primary antibodies were incubated overnight at 4 °C, and the HRP-conjugated secondary antibodies were incubated for 1–2 h at room temperature. Final detection was performed by using ECL Plus Western Blotting Detection Reagents (#WBULS0500, Millipore). Antibodies against TAZ (#4883), β-catenin (#8480), p-ATR (#2853), p-ATM (#5883), p-BRCA1 (#9009), p-CHK1 (#2348), p-CHK2 (Thr1079, #8654) were purchased from Cell Signaling Technology (Beverly, MA, USA). Antibodies against p21 (#sc-6246), hTERT (#sc-393013), Rad51C (#sc-56214) were purchased from Santa Cruz Biotechnology (Santa Cruz, CA, USA). Antibodies against TRF1 (#ab129177), TRF2 (#ab108997), POT1 (#ab124784), TIN2 (#ab197894), RAP1 (#ab175329) were obtained from Abcam (Cambridge, MA, USA). Antibodies against GAPDH (#HRP-6004), β-actin (#HRP-60008) were obtained from Proteintech Group Inc. (Wuhan, China).

### DNA/RNA isolation, RT-PCR and qPCR

For relative telomere length analysis, genomic DNA was isolated by using the Tissue DNA Purification Kit (#D3396-01, Omega) and used as the template of qRCR reaction to determine the relative telomere length.

For mRNA expression analysis (RT-PCR), total RNAs were isolated by using the RNA Fast 200 isolation kit (#220010, Feijie, Shanghai, China) and reversely transcribed into cDNA by using the cDNA Reverse Transcription Premix (#2641A, Takara) according to the manufacture’s instruction. 1 μl of cDNA was then used for qPCR analyses.

For the analysis of TERRAs expression, RNAs were isolated by using the universal RNA extraction Kit (#9767, Takara). The isolated RNAs were then reversely transcribed by using the complement telomere repeats (CCCTAA)5 as the primers to detect the levels of TERRAs.

qPCR mixtures were prepared by using SYBR qPCR Premix (#RR420L, Takara). qPCR analyses were then performed with the Bio-Rad CFX96TM Real-Time PCR detection system according to the instruction of the qPCR Premix manual. Relative telomere length was normalized to single-copy gene HBG. Fold changes were determined by the ΔΔCt method. mRNA expression was normalized to *GAPDH* levels and also determined by the ΔΔCt method. All primers are sythesized by Shenggong company (Shanghai, China), and the sequences are listed in Additional file 1: Table S2.

### Southern analysis of terminal restriction fragments

Telomere length was measured by using the Telo TAGGG Telomere Length Assay kit (#12209136001, Roche) according to the manufacturer’s protocol. Briefly, genomic DNA was digested using Hinf I and Rsa I. The terminal restriction fragments (TRFs) were separated by 0.8% agarose gel electrophoresis at 60 V for 4 h and then capillary transferred to the positively charged nylon membrane. DIG-labeled telomere-specific probe was used to hybridization with the TRFs. The DIG-specific antibody coupled to alkaline phosphate was used for chemiluminescence detection.

### Cell viability assays

Cell viability was assessed by the MTT assay. Briefly, equal amount of cells were seeded into a 48-well plate. At specific time points after seeding, 50 μl MTT (5 mg/ml) was added into the culture medium and incubated for 4 h at 37 °C, and then the mediums were removed. The generated formazan crystals were dissolved in 375 μl DMSO, and the optical absorbance values were determined at 490 nm by using a microplate reader (PerkinElmer).

### Cell cycle assay

Cells were fixed in cold 70% ethanol at 4 °C overnight. Cells were incubated with 50 μg/mL sodium citrate and 10 μg/mL RNase A in the dark for 30 min. FACS Calibur flow cytometer (BD Biosciences) was used to determine the DNA content. The data were analyzed using the MODFIT software program (Verity Software House).

### BrdU incorporation assay

BrdU assay was performed according to the protocol provided by BioLegend. Briefly, 10 µM BrdU was added to actively dividing cells for 45 min. Cells were fixed in 70% ethanol and incubated with 2N HCl and 0.1M Na_2_B_4_O_7_. Anti-BrdU antibodies (#364103, BioLegend) were then added and incubated for 20 min at room temperature. FACS analysis was performed with a Becton Dickinson Canto instrument (BD Biosciences) to determine the incorporated BrdU levels.

### Senescence-associated β-Galactosidase staining

Cell senescence was measured by using a SA-β-gal staining kit (Genemed, Beijing, China). The experiment was performed according to the product manual. Briefly,cells were fixed and stained with SA-β-gal staining solution overnight. Cells were examined under a light microscope. The number of the SA-β-gal-positive blue cells was counted, and the percentage of the SA-β-gal-positive cells versus total cells was calculated.

### Luciferase reporter assay

The 2.9 kb or 300 bp hTERT promoters upstream of the hTERT transcription start site were cloned into pGL4.17 vector which encodes the *Photinus pyralis* luciferase reporter gene luc2 (#E6721, Promega). The 2 kb Rad51C promoters upstream of Rad51C transcription start site was cloned into pGL4.17 vector. The constructed plasmids were transfected into cells together with pRL-SV40 Renila Luciferase Control Reporter Vectors (#E2231, Promega) by using Lipofectamine 2000 (#11668-019, Invitrogene). 48 h after the transfection, the cell lysates were prepared, and the luciferase activities were measured by using the Dual Luciferase Reporter Assay system (Promega) according to the instruction. The renila luciferase was the control to normalize the transcriptional activity of hTERT of Rad51C promoter fragments.

### Immunofluorescence (IF) and IF-Fluorescence in situ hybridization (IF-FISH) analyses

For IF analysis, cells were seeded on coverslips and fixed in 4% paraformaldehyde for 10 min. 0.2% Triton X-100 was used to permeabilize the membranes. Cells were then incubated with blocking solution for 1 h, followed by incubating with primary antibodies overnight at 4 °C. Antibodies against TRF2 (#ab108997, Abcam), p-BRCA1 (#9009, Cell Signaling Technology), 53BP1 (#NB100-304, Novus), γ-H2AX (#05-636, Merck Millipore Corporation), p53(#sc-126, Santa Cruz Biotechnology) were used as primary antibodies. Alexa fluor 488-labeled or Cy3-labeled secondary antibodies (Zhuangzhi Bio, China) were further incubated for another 2 h. Cells were then counterstained with DAPI and imaged with the laser scanning confocal microscope (Leica).

For IF-FISH, FISH was performed after IF staining described above. Briefly, after secondary antibodies incubation, cells were washed with PBS for 3 times. 4% paraformaldehyde containing 0.1% Triton X-100 was added to re-fix cells. Cells were then incubated with 10 mM glycine for 30 min. The telomeric PNA probes (PANAGENE) were added in a hybridization buffer containing 70% formamide, 1 mg/ml blocking reagent (Roche), 10 mM Tris–HCl pH 7.4, and denatured at 80 °C for 5 m. The probes were then hybridized in a humidified chamber for 2 h at room temperature. After washing the coverslips with wash solution 1 (containing 70% formamide, 0.1% BSA, 10 mM Tris–HCl pH7.4) and wash solution 2 (containing 100 mM Tris–HCl pH7.4, 150 mM NaCl, Tween-20), cells were counterstained with DAPI and imaged with the laser scanning confocal microscope (Leica).

### Telomerase activity assay

Telomerase activity was measured by using TRAPEZE Telomerase Detection Kit (#S7700, Millipore) according to the manufacturer’s protocol. Briefly, 10^6^ cells were resuspended in 200 μL of 1 × CHAPS lysis buffer and incubated on ice for 30 min. Cell extracts were then diluted in a ratio of 1:5. 2 μl of the diluted cell extracts together with TRAP Reaction Buffer, dNTP Mix, TS Primer, TRAP Primer Mix, and Taq polymerase were prepared and subjected to PCR reaction. The PCR program is designed following the manufacturer’s instruction. The PCR products were separated with 15% non-denaturing PAGE and detected with silver staining.

### Chromatin-IP (ChIP) assay

ChIP was performed following the manufacture’s instruction of MegnaChIP A/G kit (#17-10085, Merk). Briefly, 5 × 10^6^ cells were cross-linked with 1% formaldehyde for 10 min at room temperature, and then quenched with glycine for another 5 min. Cells were lysed and sonicated by using Bioruptor (#UCD-200, Diagenode) with a condition of “30 s ON and 30 s OFF” for 8 cycles at high power setting. Sheared chromatin was incubated with protein A/G magnetic beads and antibodies against p53 (#sc-126X, Santa Cruz Biotechnology) or TEAD4 (ab58310, Abcam) and a mouse or rabbit IgG at 4 °C over night. Genomic DNA was purified after reverse-crosslinking. ChIP DNA was further analyzed by qPCR with primers specific to the p53 binding region of 13q-TERRA promoter or Tead4 binding region of Rad51C promoter. The sequences are listed. 13q-ChIP-Forward: 5′-GACAAGCCCCAGCCCATA-3′, 13q-ChIP-Reverse: 5′-GGGATAGTTAAGGAAGGATCTCCAA-3′, Rad51C-ChIP-Forward: 5′-CCGTTACGCTCACTACTCCC-3′, Rad51C-ChIP-Reverse: 5′-CGGAGACCATAGGACGGAGA-3′.

### Statistical analysis

Statistic analysis was performed using SPSS Version 18.0. All results are presented as mean ± SEM. Shapiro–Wilk test was used for normality test. The data were considered to be normally distributed while *P* > 0.1, and all data were normally distributed. Statistical analyses were performed with unpaired Student's *t*-test between two groups and one-way ANOVA followed by Tukey’s multiple-comparisons for multiple groups. Two-way ANOVA followed by Bonferroni post test were used for MTT experiment analysis. All data were derived from at least three independent experimental replicates. *P* value < 0.05 was considered as statistically significant.

## Results

### Knockdown of TAZ inhibits cell proliferation and causes cell senescence in TNBC cells

**Fig. 1:**
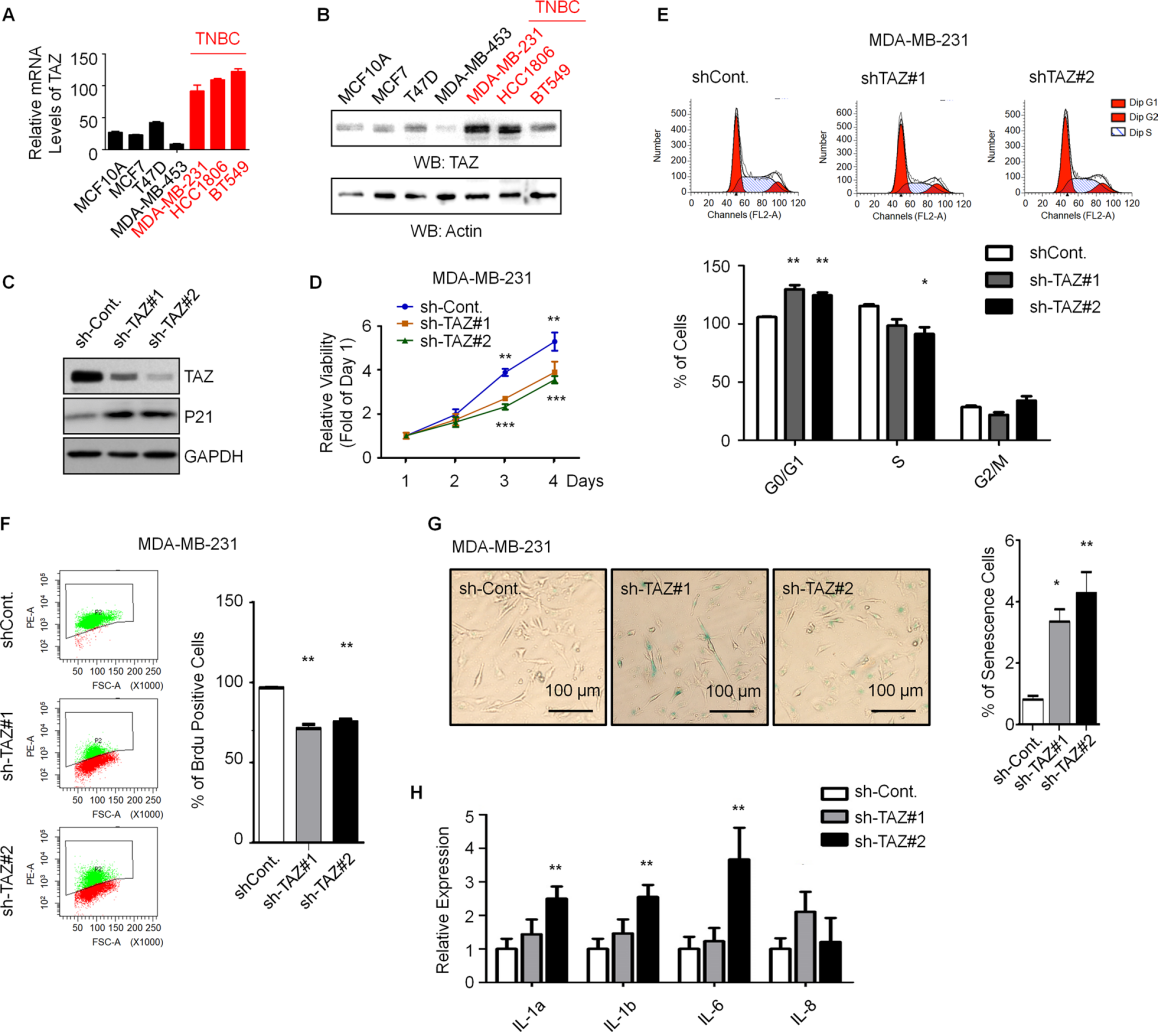
Knockdown of the expression of TAZ causes cell senescence in TNBC cells. **A** qPCR analysis in different breast cancer cell lines to indicate the mRNA levels of TAZ. **B** Western blot analysis in different breast cancer cell lines to indicate the protein levels of TAZ. Quantification of Western blots is shown in Additional file 1: Fig. S5A. **C** Western blot analysis in a control shRNA-transfected (sh-Cont.) and two separate TAZ-specific shRNAs-transfected (sh-TAZ) MDA-MB-231 cells to indicate the protein levels of TAZ and P21. Quantification of Western blots is shown in Additional file 1: Fig. S5B. **D** Cell viability assays were performed in the indicated MDA-MB-231 cells. E, Cell cycle assays were performed in the indicated MDA-MB-231 cells. **F** BrdU incorporation assays were performed in the indicated MDA-MB-231 cells. **G** Senescence-associated β-galactosidase activity staining analyses were performed in the indicated MDA-MB-231 cells. **H** qPCR analysis in the indicated MDA-MB-231 cells to indicated the mRNA levels of multiple SASP (senescence-associated secretory phenotype) marker genes. Data are presented as mean ± SEM. At least three repeats were carried out for each test. The p values of cell viability assays were determined by two-way ANOVA followed by Bonferroni post test. All other p values were determined by one-way ANOVA followed by Tukey’s multiple-comparisons. NS, not significant, *p < 0.05, **p < 0.01, ***p < 0.001

To know the potential roles of TAZ in the regulation of TNBC, we firstly examined the expressional levels of TAZ in both TNBC and non-TNBC. The bioinformatic analyses revealed that the mRNA levels of TAZ are severely higher in both TNBC patients and cell lines than that in non-TNBC (Additional file 1: Fig. S1A and B). These results are consistent to the previous studies that TAZ mRNA and protein expression are preferentially higher in TNBC than in other non-TNBC subclasses [16, 17]. Next, we verified the expressional changes of TAZ between TNBC and non-TNBC by real-time RT-PCR and Western blot analyses in several breast cancer cell lines. In accordance with the data from the above bioinformatic analyses, we found that both the mRNA and protein levels of TAZ are much higher in TNBC cell lines than that in non-TNBC (Fig. 1A, B). Furthermore, copy number variation (CNV) analysis revealed that gain of copy numbers of TAZ gene occurs more frequently in TNBC patients than that in non-TNBC patients (Additional file 1: Fig. S1C). Together, our results indicated that the expressional levels of TAZ are highly upregulated in TNBC. To understand the biological impacts of TAZ in TNBC, we knocked down the expression of TAZ in MDA-MB-231 TNBC cells by distinct shRNAs (Fig. 1C). We found that loss of TAZ significantly prohibits the proliferation of MDA-MB-231 cells (Fig. 1D). The data of cell cycle assay showed that loss of TAZ results in an obvious accumulation of cells in the G0/G1-phase and a decrease of cells in the S-phase. (Fig. 1E). BrdU incorporation assays further confirmed the observation that downregulation of TAZ causes an impaired proliferation activity (Fig. 1F). Additionally, we also found that depletion of TAZ causes significant higher levels of senescent cells (Fig. 1G), upregulation of p21 (Fig. 1C), and increased expressions of senescence-associated cytokines (Fig. 1H), suggesting that an elevated cell senescence is occurred in TAZ-depleted TNBC cells. Taken together, these data suggested that knockdown of TAZ expression inhibits cell proliferation and triggers cell senescence in TNBC cells.

### TAZ is essential for telomere maintenance in TNBC cells

**Fig. 2:**
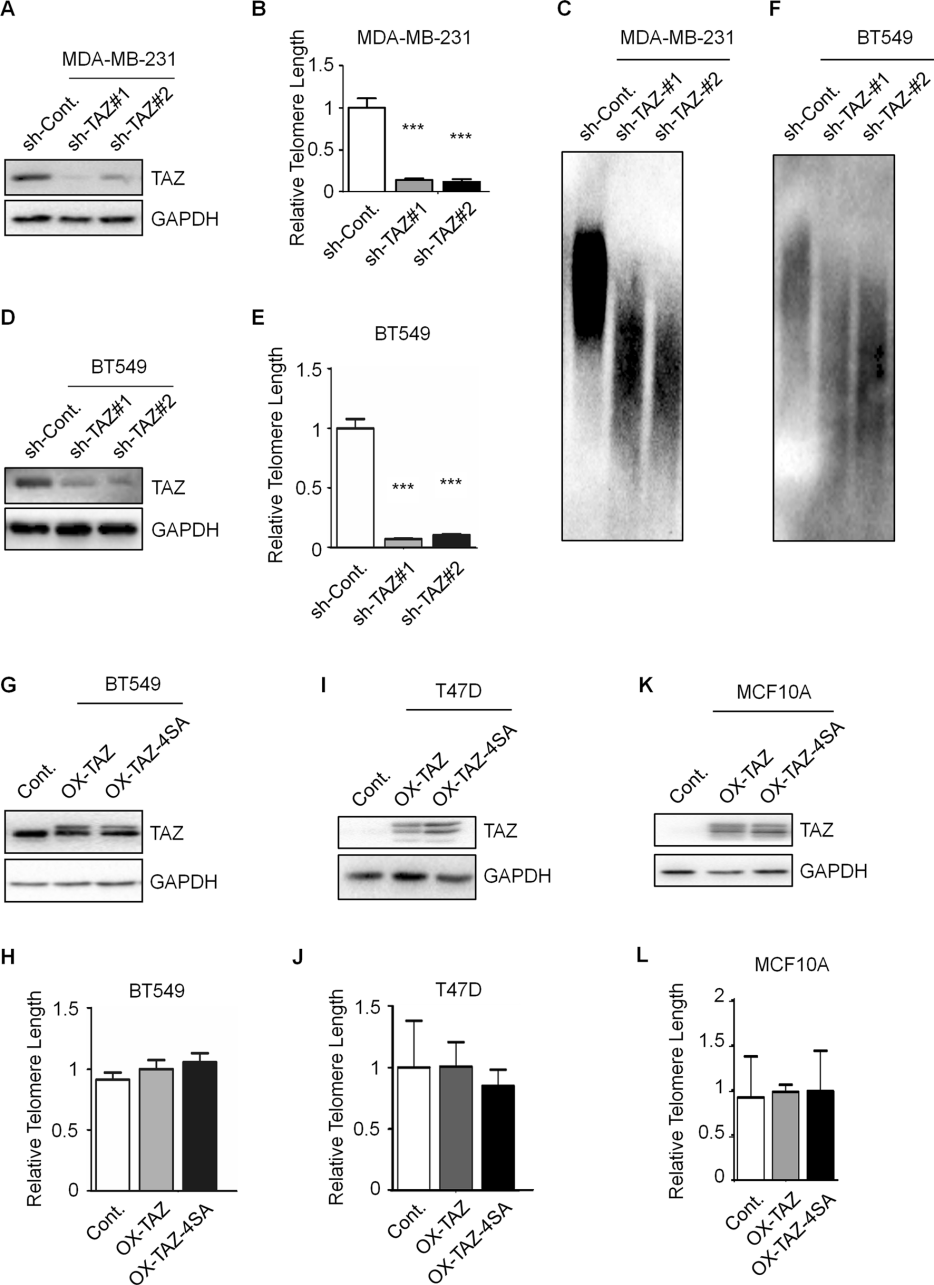
TAZ is essential for telomere maintenance in TNBC cells. **A** Western blot analysis in the indicated MDA-MB-231 cells to indicate the depletion efficiency of TAZ expression. Quantification of Western blots is shown in Additional file 1: Fig. S5C. **B** Telomere-specific qPCR analysis in the indicated MDA-MB-231 cells to determine the relative telomere length. **C** Southern analysis of TRFs in the indicated MDA-MB-231 cells to determine the relative telomere length. **D** Western blot analysis in the indicated BT549 cells to indicate the protein levels of TAZ. Quantification of Western blots is shown in Additional file 1: Fig. S5D. **E** Telomere-specific qPCR analysis in the indicated BT-549 cells to analyze the relative telomere length. **F** Southern analysis of TRFs in the indicated BT549 cells to determine the relative telomere length. **G** Western blot analysis in BT549 cells overexpressing wild type TAZ and its activated mutant (TAZ-4SA) with antibodies as indicated. Quantification of Western blots is shown in Additional file 1: Fig. S5E. **H** Telomere-specific qPCR analysis to determine the relative telomere length in the indicated BT549 cells. **I** Western blot analysis in T47D cells overexpressing wild type TAZ and its activated mutant (TAZ-4SA) with antibodies as indicated. **J** Telomere-specific qPCR analysis to determine the relative telomere length in the indicated T47D cells. **K** Western blot analysis in MCF10A cells overexpressing wild type TAZ and its activated mutant (TAZ-4SA) with antibodies as indicated. **L** Telomere-specific qPCR analysis to determine the relative telomere length in the indicated MCF10A cells. Data are presented as mean ± SEM. At least three repeats were carried out for each test. The *p* values were determined by one-way ANOVA followed by Tukey’s multiple-comparisons. NS, not significant, *p < 0.05, **p < 0.01, ***p < 0.001

It has been reported that cell senescence is tightly related to shortened telomeres [18]. Together with the findings that loss of TAZ results in cell senescence in TNBC cells (Fig. 1C–H), we therefore suspected that TAZ may affect telomere length in TNBC cells. Intriguingly, telomere-specific qPCR analysis revealed that knockdown of TAZ expression in MDA-MB-231 and BT549 TNBC cells (Fig. 2A, D) leads to a significant decrease of telomere length in both cell lines (Fig. 2B, E). We further confirmed the shortened telomere length in TAZ-depleted cells with Southern analysis of terminal restriction fragments. The terminal restriction fragments were generated by digesting genomic DNA with Hinf I and Rsa I, and detected with telomere specific probe. Knockdown of TAZ causes shorter telomere length characterized by the decrease in TRF length (Fig. 2C, F). We next investigated the effect of TAZ overexpression on telomere length. The plasmids encoding a Flag-tagged wild-type TAZ (Flag-TAZ) and a Flag-tagged TAZ mutant that permanently unphosphorylated (FLAG-TAZ-4SA), which indicates the constitutively activated (CA) form of TAZ, were transfected into BT549 cells (Fig. 2G). Telomere length was then tested, and our results revealed that no obvious changes of the telomere length was observed in both types of TAZ-overexpressing cells (wild-type and the CA mutant) (Fig. 2H). We also overexpressed the wild-type and the CA mutant TAZ in T47D cells which are non-TNBC cells with low TAZ expression and in MCF10A cells which are non-transformed mammary epithelial cells (Fig. 2I, K). No obvious changes of telomere length was observed (Fig. 2J, L). These results suggested that the inhibition of TAZ shortens telomeres in TNBC cells, while the activation of TAZ has no effect on telomere length in both TNBC and non-TNBC cells.

### Knockdown of TAZ upregulates the expression of hTERT in TNBC cells

**Fig. 3:**
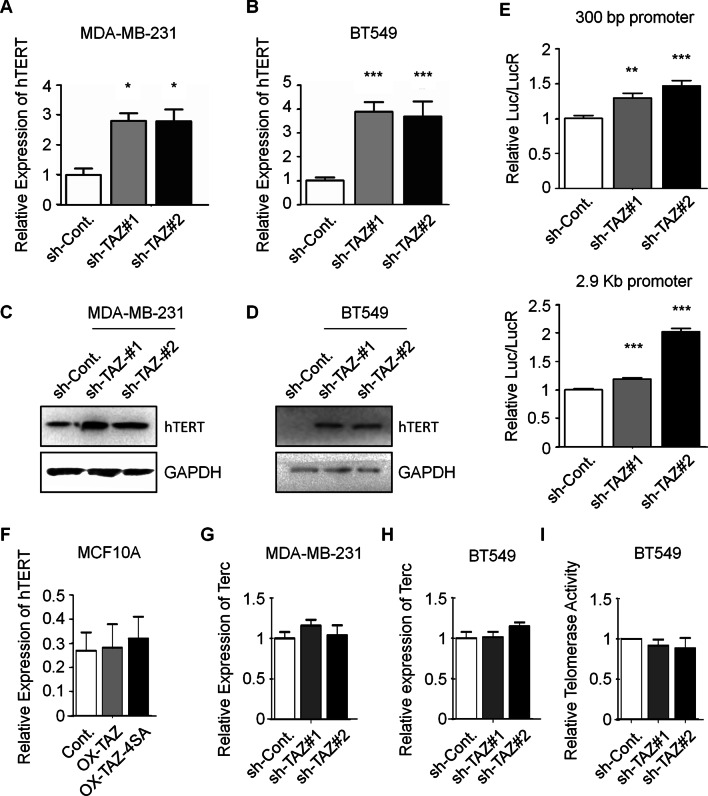
Depletion of TAZ increases the expression of hTERT in TNBC cells. **A** and **B** qPCR analyses to indicate the mRNA levels of hTERT in indicated MDA-MB-231 and BT549 cells. **C** and **D** Western blot analyses in the indicated MDA-MB-231 and BT549 cells to examine the protein levels of hTERT. Quantification of Western blots is shown in Additional file 1: Fig. S5F. **E** Luciferase reporter assays were performed to test the activities of 300 bp and 2.9 kb hTERT promoter fragments in the indicated MDA-MB-231 cells. **F** qPCR analyses to indicate the mRNA levels of hTERT in indicated MCF10A cells. **G** and **H** qPCR analyses to indicate the mRNA levels of Terc in indicated MDA-MB-231 and BT549 cells. **I** TRAP analysis to measure telomerase activities in BT549 cells transfected as indicated. Data are presented as mean ± SEM. At least three repeats were carried out for each test. The p values were determined by one-way ANOVA followed by Tukey’s multiple-comparisons. NS, not significant, *p < 0.05, **p < 0.01, ***p < 0.001

Previous studies indicated that hTERT is essential for the maintenance of telomere length [19]. Together with our findings that loss of TAZ results in significantly shortened telomeres in TNBC cells (Fig. 2), we therefore attempted to test whether TAZ affects the expression of hTERT. To our surprise, not decrease, our results revealed that loss of TAZ results in elevated mRNA and protein levels of hTERT in both MDA-MB-231 and BT549 TNBC cells (Fig. 3A–D). It has been reported that the ~ 2900 bp upstream region containing ~ 300 bp core nucleotides functions as the promoter of hTERT [20, 21]. We therefore cloned these two nucleotides regions into a luciferase reporter construct and performed dual luciferase reporter assays to indicate the effect of TAZ on hTERT promoter activity. In accordance to the effect on hTERT expression, our results indicated that loss of TAZ promotes the luciferase activity suggesting that an elevated hTERT promoter activity occurs under TAZ depletion (Fig. 3E). Overexpression of wild-type TAZ and its CA mutant result in no obvious change of hTERT level in MCF10A non-transformed mammary epithelial cells (Fig. 3F). Terc is an RNA molecule that provides the template for telomerase to add repeated DNA sequence to the ends of telomeres. We therefore tested the levels Terc after TAZ depletion, and no obvious changes was observed in TNBC cells (Fig. 3G, H). In addition, our telomeric repeat amplification protocol (TRAP) assay indicated no significant change of telomerase activity in TAZ-depleted cells (Fig. 3I), further supporting that the changes of hTERT are not the cause of TAZ depletion-induced telomere shortening. Together, these data indicated that knockdown of TAZ upregulates the expression of hTERT in TNBC cells.

### TAZ-depleted cells in early passages show shortened telomeres and no changes of hTERT expression

Accidentally, we found that knocking down the expression of TAZ with two TAZ-specific siRNAs shows a different effect on hTERT mRNA levels compared with knocking down by using TAZ-specific shRNA. In contrast to the effect of TAZ-specific shRNA (Fig. 3A–D), knockdown of TAZ expression in TNBC cells with TAZ-specific siRNAs transfected for 48 h (Additional file 1: Fig. S2A and B) does not show obvious effect on hTERT mRNA levels (Additional file 1: Fig. S2C and D) and protein levels (Additional file 1: Fig. S2E and F). We therefore tested whether the siRNA-mediated TAZ knockdown can also result in shortened telomere length as detected in TNBC cells with TAZ knockdown by using TAZ-specific shRNAs (Fig. 2). Intriguingly, depletion of TAZ by siRNAs indeed leads to a shortened telomere length phenotype (Additional file 1: Fig. S2G–J), further supporting that hTERT is likely not involved in TAZ-mediated telomere length regulation.

As cells transfected with siRNAs were usually harvested for examination at 48 h after transfection (short-term), while cells transfected with shRNAs were passaged for more than six passages (long-term) when subjected to the related analyses, we therefore speculated that long-term knockdown of TAZ may cause more extreme telomere shortening. We next examined the dynamics of telomere length changes after TAZ knockdown during different passages. As expected, telomere length indeed shows more severe shortening phenotype during prolonged passages (Additional file 1: Fig. S2K), and short time (48 h) depletion of TAZ with shRNAs also shows no obvious effect on hTERT mRNA levels, similar to the effect of siRNAs (Additional file 1: Fig. S2L).

### Knockdown of TAZ results in telomere deprotection and causes DDR

**Fig. 4:**
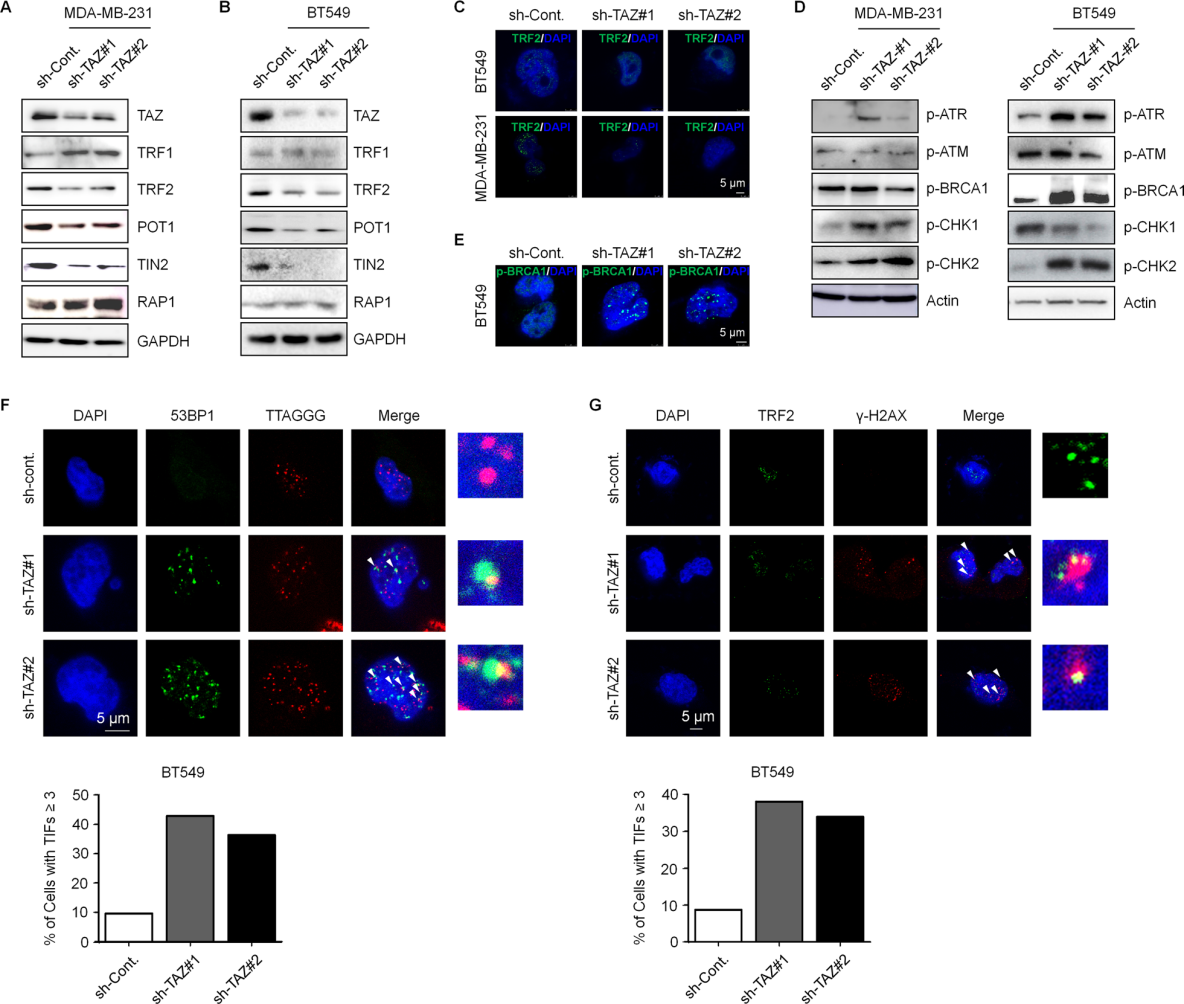
Knockdown of TAZ results in the deprotection of telomeres and the activation of DDR. **A** Western blot analysis of shelterin proteins in TAZ-depleted MDA-MB-231 cells with long-term approaches (infected with shRNA and cultured > 6 passages). Quantification of Western blots is shown in Additional file 1: Fig. S5G. **B** Western blot analysis of shelterin proteins in long-term TAZ knockdown (infected with shRNA and cultured > 6 passages) BT549 cells. Quantification of Western blots is shown in Additional file 1: Fig. S5H. **C** Immunofluorescence assay of TRF2 in BT549 and MDA-MB-231 cells treated as indicated. **D** Western blot analysis of the DDR pathway proteins in MDA-MB-231 and BT549 cell with long-term TAZ knockdown. Quantification of Western blots is shown in Additional file 1: Fig. S5I. **E** Immunofluorescence assay of p-BRCA1 in BT-549 cells with long-term TAZ knockdown. **F** IF-FISH assays stained with 53BP1 antibody and Cy3-conjugated telomere PNA probes were performed in the indicated BT549 cells. Co-localizing events indicate telomere dysfunction induced foci (TIFs). The percentage of cells with more than 3 TIFs were analyzed. 100 cells were counted for each experiment. **G** Immunofluorescence assays of TRF2 and γ-H2AX in the indicated BT549 cells. Co-localizing events indicate telomere dysfunction induced foci (TIFs). The percentage of cells with more than 3 TIFs were analyzed. 100 cells were counted for each experiment

As mentioned above, shelterin complex is tightly involved in the regulation of telomere length by directly binding to telomere DNA to protect telomeres [22]. Therefore, we examined the effect of TAZ depletion by both short-term and long-term approaches on the protein levels of shelterin components. Intriguingly, similar to the effect on hTERT expression, short-term knockdown and long-term knockdown of TAZ expression also showed different impacts on the protein levels of shelterin components. We found that short-term depletion of TAZ showed no obvious effect on the protein levels of the shelterin components (Additional file 1: Fig. S3). However, by contrast, long-term knockdown of TAZ expression resulted in significant decreases of the protein levels of most of the shelterin components except TRF1 and RAP1 (Fig. 4A, B), suggesting that an impaired shelterin complex organization is likely occurred in long-term TAZ-depleted TNBC cells. To further confirm this observation, we performed an IF assay to detect the levels of TRF2. The levels of TRF2 are strongly decreased in long-term TAZ-knocked down TNBC cells (Fig. 4C).

As it has been reported that shelterin complex prevents DDR activation through protecting telomeres[23], and our findings that long-term loss of TAZ downregulates the protein levels of multiple shelterin components (Fig. 4A, B), we therefore examined the activity of the DDR pathway upon knockdown of TAZ expression. As expected, most of the molecular markers of the DDR pathway are upregulated after long-term depletion of TAZ expression in TNBC cells suggesting that knockdown of TAZ expression with long-term approaches activates the DDR pathway (Fig. 4D). Moreover, our IF analysis with phosphorylated BRCA1 antibodies (anti-p-BRCA1) also supports this observation as elevated p-BRCA1 signals were detected after TAZ depletion in BT549 cells with a long-term approach (Fig. 4E). We next checked the telomere-induced DNA damage foci (TIF) in both TAZ-deficient and control cells. We found that the percentage of cells with more than 3 TIFs are elevated in TAZ-depleted cells (Fig. 4F, G). Taken together, these data suggested that long-term depletion of TAZ affects shelterin complex and activates the DDR pathway. Therefore, TAZ positively regulates telomere integrity in TNBC cells.

### Knockdown of TAZ activates TERRAs transcription

To explore the potential mechanisms of how TAZ affects telomere length, we determined to test the levels of TERRAs expression, as TERRAs have been reported to regulate telomere length [15, 24]. Therefore, we firstly examined whether loss of TAZ affects the expression levels of different TERRA family members. We detected that depletion of TAZ with both short-term and long-term approaches in TNBC cells all result in upregulated expressions of multiple members of TERRAs (Additional file 1: Fig. S4A–D). These results suggested that loss of TAZ promotes the expressions of TERRA family members, which occurs in both short-term and long-term TAZ depletion conditions, and may contribute to the detected telomere length shortening induced by TAZ depletion.

### TAZ maintains telomere length through contributing to Rad51C Expression

**Fig. 5:**
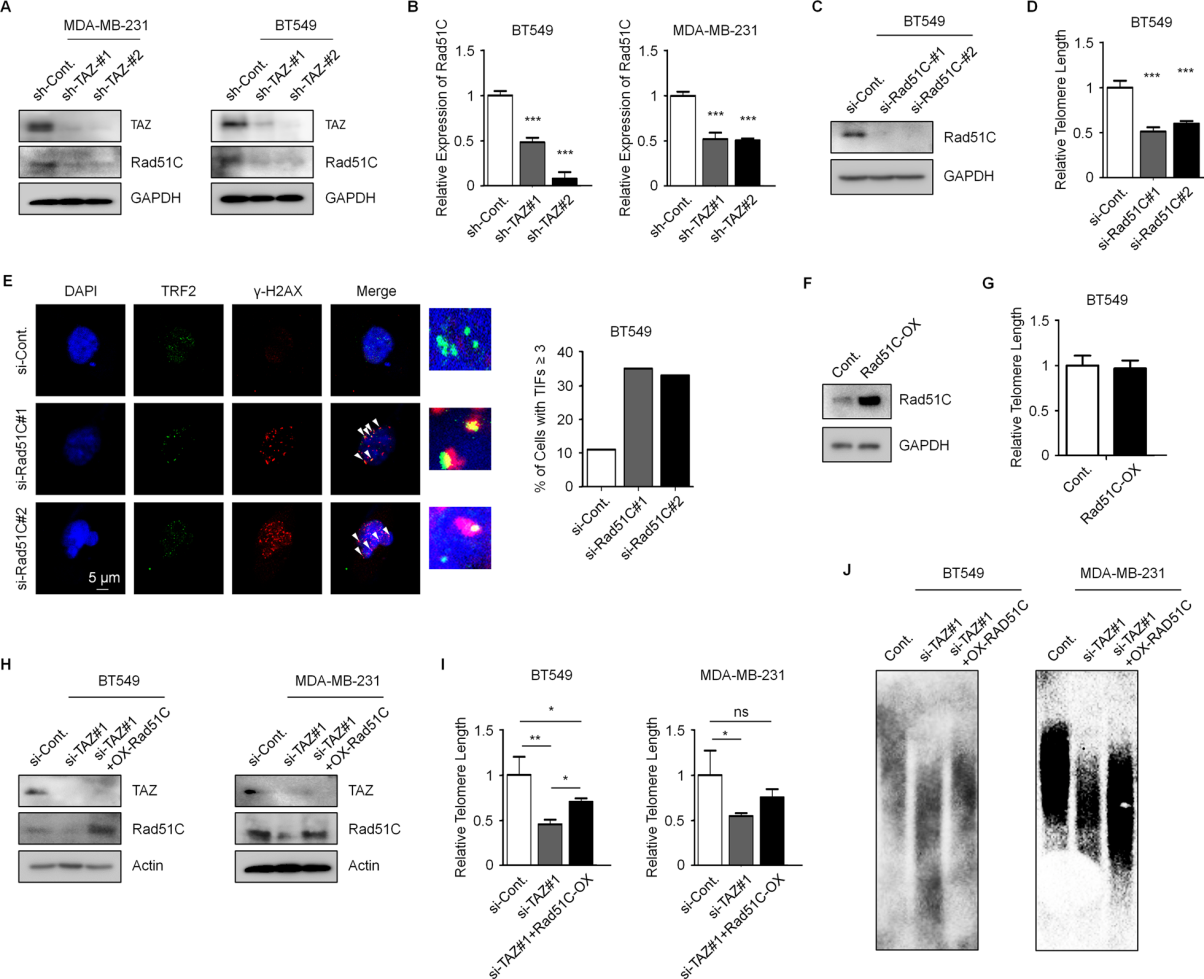
Knockdown of TAZ inhibits Rad51C transcription, and TAZ maintains telomere length through Rad51C.** A** Western blot analysis of TAZ and Rad51C protein levels in MDA-MB-231 and BT549 cells as indicated. Quantification of Western blots is shown in Additional file 1: Fig. S5J. **B** qPCR analyses to determine the mRNA levels of Rad51C in MDA-MB-231 and BT549 cells as indicated. **C** Western blot analysis in BT549 cells treated as indicated to examine the protein levels of Rad51C. Quantification of Western blots is shown in Additional file 1: Fig. S5K. **D** Telomere-specific qPCR analysis to determine the relative telomere length in the indicated BT549 cells. **E** Immunofluorescence assays of TRF2 and γ-H2AX in the indicated BT549 cells. Co-localizing events indicate telomere dysfunction induced foci (TIFs). The percentage of cells with more than 3 TIFs were analyzed. 100 cells were counted for each experiment. **F** Western blot analyses in BT549 cells as indicated. Quantification of Western blots is shown in Additional file 1: Fig. S5L. **G** Telomere-specific qPCR analysis to determine the relative telomere length in the indicated BT549 cells. **H** Western blot analyses in BT549 cells and MDA-MB-231 treated as indicated to examine the protein levels of TAZ and Rad51C. Quantification of Western blots is shown in Additional file 1: Fig. S5M. **I** Telomere-specific qPCR analysis to determine the relative telomere length in the indicated BT549 and MDA-MB-231 cells. **J** Southern analysis of TRFs in the indicated BT549 and MDA-MB-231 cells to determine the relative telomere length. Data are presented as mean ± SEM. At least three repeats were carried out for each test. Statistical analyses were performed with unpaired Student's *t*-test between two groups and one-way ANOVA followed by Tukey’s multiple-comparisons for multiple groups. NS, not significant, *p < 0.05, **p < 0.01, ***p < 0.001

**Fig. 6:**
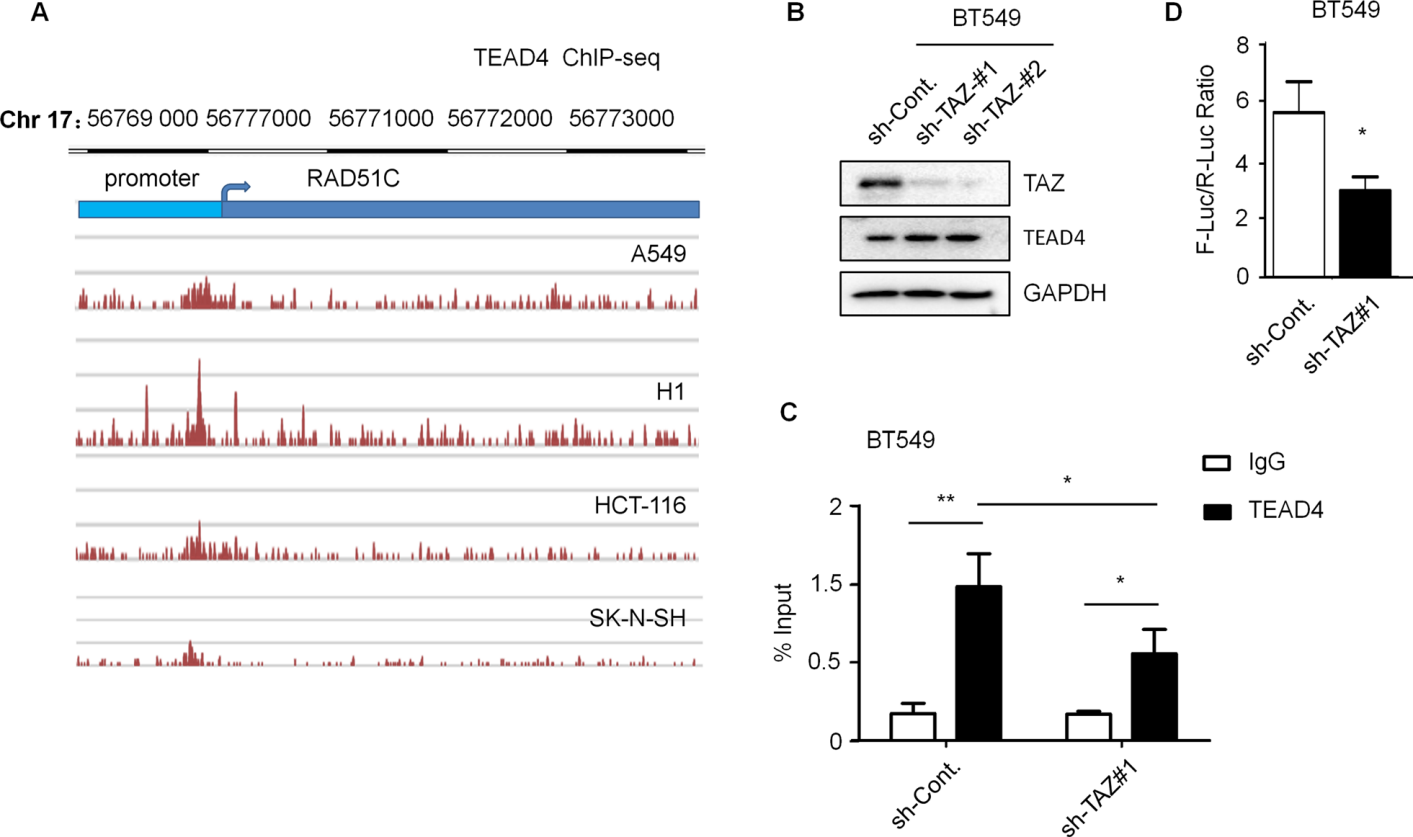
Rad51C is a direct YAP/TAZ-TEAD target gene. **A** TEAD4 binding signals in the promoter of Rad51C from ENCODE ChIP-seq database (https://www.encodeproject.org) were analyzed from A549 (lung cancer), H1 (human embryonic stem cells), HCT-116 (colon cancer), MCF-7 (breast cancer), and SK-N-SH (neuroblastoma) cells. **B** Western blot analysis of TAZ and TEAD4 protein levels in BT549 cells as indicated. Quantification of Western blots is shown in Additional file 1: Fig. S5N. **C** ChIP analyses in the indicated BT549 cells were performed to determine the enrichment of TRAD4 on the Rad51C promoter region. IgG was used as a negative control. **D** Luciferase reporter assays were performed to test the activities of Rad51C promoter fragments in the indicated BT549. Data are presented as mean ± SEM. At least three repeats were carried out for each test. The *p* values were determined by Student's t-test. NS, not significant, *p < 0.05, **p < 0.01, ***p < 0.001

Rad51C participates in homologous recombination. Loss of Rad51C in both telomerase positive and negative MEF cells causes rapid loss of telomere sequences and breakage of telomere integrity [25]. This Rad51C depletion-induced phenotype in telomere is similar to what we observed in TAZ-knocked down cells. We therefore tested Rad51C levels in TAZ-depleted and control cells. Loss of TAZ results in reduced mRNA and protein levels of Rad51C in both MDA-MB-231 and BT549 TNBC cells (Fig. 5A, B). In addition, knocking down the expression of Rad51C in BT549 cells (Fig. 5C) leads to a significant decrease of telomere length (Fig. 5D) and increased cells with more than 3 telomere-induced DNA damage foci (Fig. 5E). Similar with TAZ overexpression, increased Rad51C expression showed no obvious changes in telomere length (Fig. 5F, G). Importantly, the telomere shortening phenotype caused by TAZ depletion is rescued by Rad51C overexpression in both MDA-MB-231 and BT549 cells (Fig. 5H–J). Together, these data suggested that loss of TAZ inhibits the transcription of Rad51C and further destroys the telomere length homeostasis and telomere integrity. Notably, overexpression of Rad51C in TAZ-depleted cells did not fully restore the telomere length to the levels of control cells (Fig. 5I, J), suggesting that additional mechanisms may also be involved in TAZ-regulated telomere length dynamics. As previously observed, increased levels of TERRAs may be an alternative mechanism causing shortened telomeres in TAZ-deficient cells. TAZ is a transcriptional co-activator that regulates transcription of target genes by binding to transcriptional factors [3]. TEAD family members (TEAD1-4) are the most common TAZ binding partners [26]. We analyzed the TEAD4 chromatin immunoprecipitation followed by high-throughput sequencing (ChIP-seq) datasets with the ENCODE database (https://www.encodeproject.org) to determine whether TAZ-TEAD directly regulate Rad51C transcription. We found that, in different types of cells (A549-lung cancer, H1-human embryonic stem cells, HCT-116-colon cancer, MCF-7-breast cancer, SK-N-SH-neuroblastoma), TEAD4 directly binds to the promoter regions of RAD51C gene (Fig. 6A). However, loss of TAZ expression did not affect the TEAD4 protein levels (Fig. 6B). We next performed ChIP-qPCR analyses in BT549 cells to validate the binding of TEAD4 to RAD51C promoter. We found that TEAD4 is indeed enriched on the promoter region of the Rad51C gene, and the binding is reduced by knocking down the expression of TAZ (Fig. 6C). RAD51C promoter luciferase assays also showed a reduction of luciferase activity in TAZ-depleted cells (Fig. 6D). Together, these results provide evidence that a TAZ-TEAD transcriptional program regulates Rad51 expression in TNBC cells.

**Fig. 7:**
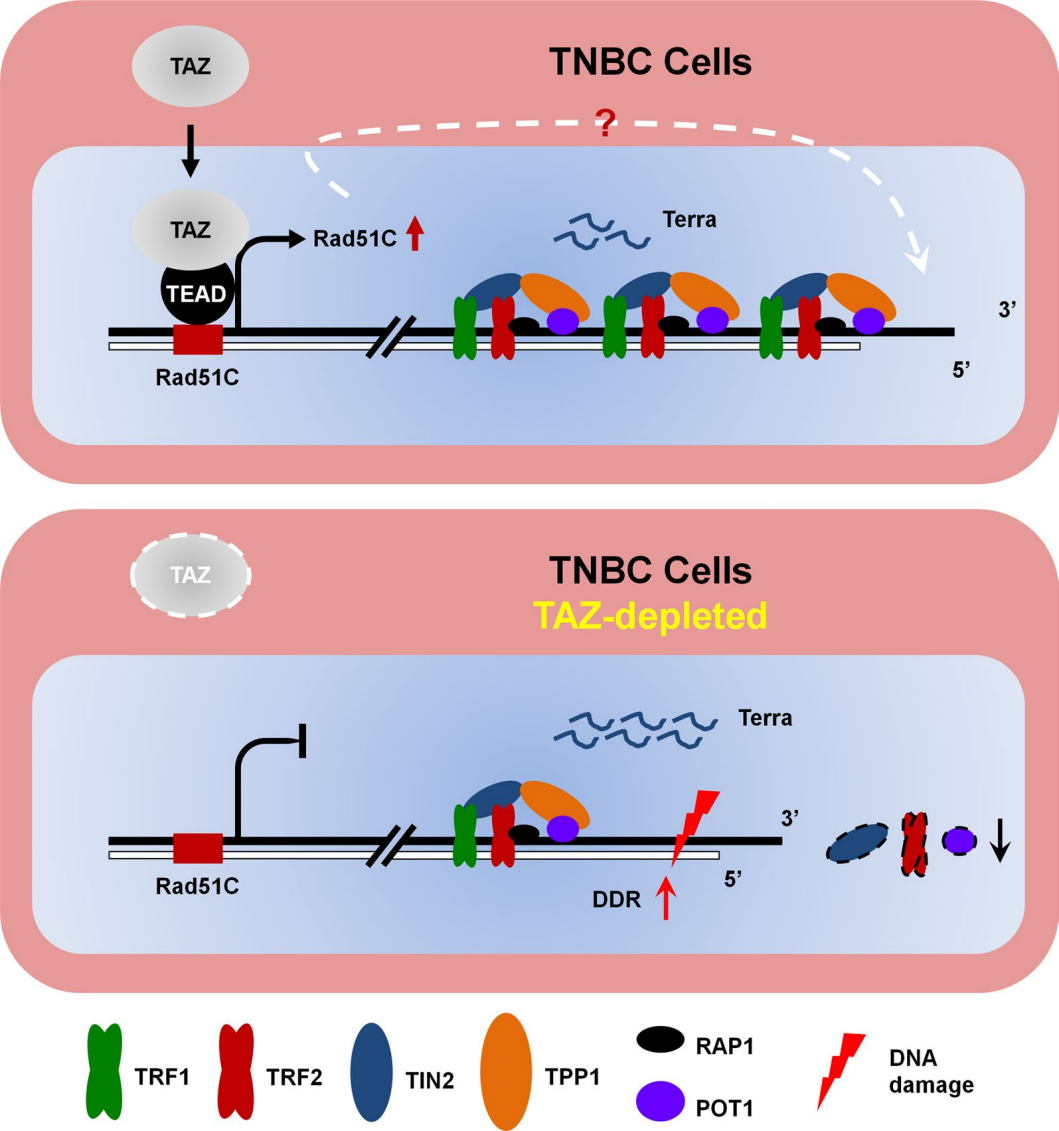
Working model of TAZ regulating telomere length in TNBC cells. In TAZ-knocked down TNBC cells, the binding of TAZ-TEADs to the promoter of Rad51C was decreased, and the transcription of Rad51C was further impaired. Lack of Rad51C causes the telomere shortening and impaired telomere functions. The protein levels of shelterin components is decreased, along with increased telomere-induced DNA damage foci (TIF) and DDR activation. Moreover, the increased levels of TERRAs in TAZ-deficient cells may also contribute to the observed telomere loss phenotype

From current study, we identified that, in TNBC cells, TAZ maintains telomere length by acting as a transcriptional co-activator of Rad51C. Knockdown of TAZ expression abolishes TAZ-TEAD binding to the promoter of Rad51C, and further impairs Rad51C transcription. Lack of Rad51C causes the telomere shortening and impaired telomere functions. The protein levels of shelterin components is decreased, along with increased telomere-induced DNA damage foci (TIF) and DDR activation. Moreover, high levels of TERRAs in TAZ-deficient cells may also contribute to the observed telomere loss phenotype (Fig. 7).

## Discussion

In this study, we examined the function of TAZ in TNBC cells and found, for the first time, that TAZ is involved in the regulation of telomere length in TNBC cells. Recently, increasing studies indicated the regulatory relationship between TAZ and cell senescence. A previous report indicated that YAP/TAZ inhibition caused a senescence-like phenotype probably through the regulation of nucleotide metabolism [27]. Loss of TAZ activity induced a senescence-like phenotype in mammary tumor-derived cells in which TAZ was highly expressed [28]. In addition, knockdown of TAZ expression induced cell senescence in a p53-dependent manner in normal human fibroblasts [29]. Consistent to previous reports, our data revealed that knockdown of TAZ expression alone is sufficient to cause cell senescence in TNBC cells. In addition, obvious shortened telomeres were parallely observed in TAZ-depleted TNBC cells. These observations are consistent with previous reports that extremely shortened telomeres can result in cell cycle arrest, cell senescence, and failures in stem cell maintenance in lung and bone marrow [11, 30, 31]**.**

Telomerase is the main modulator in most cancers to maintain telomere length [12]. To our surprise, rather than decrease, the catalytic subunit of telomerase, hTERT shows an obvious activation when TAZ was depleted. Our data also indicated that the expression of hTERT shows no obvious difference in early passages of TAZ-knocked down TNBC cells, although telomere shortening has been occurred. It has been reported that long telomeres repress endogenous expression of hTERT through forming repressive chromatin loops in telomerase-positive cancer cells [32]. Therefore, it is possible that, in late passages of TAZ knockdown cells, extremely shortened telomeres cannot hold the repressive chromatin loops, further showing a TPE-OLD (telomere position effect—over long distance) effect. Therefore, an elevated hTERT expression was observed.

As mentioned above, TERRAs are another group of key regulators of telomere length and integrity. Unlike the properties of hTERT and shelterin expression in TAZ-knocked down TNBC cells, the expression of TERRAs increases in both early and late passages of TAZ-depleted TNBC cells. The steady levels of hTERT or shelterin proteins cannot explain the shortened telomeres observed in the early passages of TAZ-depleted cells. We suspected that the excessive levels of TERRAs caused by TAZ depletion may contribute to telomere shortening observed in TAZ-depleted TNBC cells. Supporting to our hypothesis, several reports indicated that TERRAs promote telomere shortening in human cells [15]. TERRAs inhibit telomerase activity by acting as competitive inhibitors of telomeric DNA by pairing with telomerase Terc RNAs [33, 34]. Therefore, it is possible that our findings that no changes of telomerase activity but significant hTERT overexpression is the consequence of the high levels of TERRAs. Increasing studies indicated that TERRAs participate in the regulation of telomeres’ function and homeostasis [24, 35]. In addition, TERRAs also promote Exo-1-dependent resection of telomeres by interacting with Ku70/80 dimer [36], and inhibit heterochromatin formation to promote telomere shortening [37].

Shelterin complexes protect telomeres from unwanted DNA damage. Loss of shelterin proteins causes uncapped telomeres and telomeric DNA damage, further followed by activations of the DDR pathway and cell senescence [38, 39]. Shelterin proteins also participate in the regulation of telomere length [22]. TRF2 acts as a negative regulator of telomere length [40]. POT1 was reported to maintain telomere-length by facilitating telomerase elongation [41]. The missense point mutation of TIN2 is tightly related to progressive telomere shortening [42]. Our results revealed that long-term depletion of TAZ results in significant reductions of shelterin proteins (including TRF2, POT1 and TIN2), together with an increase of TIFs and the activation of the DDR pathway (Fig. 4). However, in the early passages of TAZ-depleted cells (48 h short-term depletion of TAZ), the levels of shelterin proteins are not changed, although the telomeres have been shortened (Additional file 1: Fig. S3). Therefore, this result excluded the possibility that the observed TAZ-regulated telomere shortening phenotype is achieved through shelterin proteins. We suspected that the observed decreases of shelterin proteins in the late passages of TAZ-depleted cells (long-term depletion of TAZ) might be the consequence of extreme telomere shortening [22, 40, 43, 44].

Rad51C is a member of the Rad51 family and regulates homologous recombination (HR) through multiple pathways [45]. Rad51C, by cooperating with distinct partners, forms two Rad51 paralog complexes: Rad51B-Rad51C-Rad51D-XRCC2 (BCDX2) and Rad51C-XRCC3 (CX3). These two Rad51 complexes facilitate homologous recombination at different stages. BCDX2 complex is responsible for the recruitment of Rad51 and the stabilization of the complex at the early stage of HR [46]. CX3 complex is involved in the resolution of HR intermediary structures at the late stage of HR [47]. The G-rich repetitive telomeric sequences and the specific structures of telomeres hamper the formation of replication fork during telomere replication. The stalled replication forks and their subsequent collapse further cause rapid telomeres deletion [48, 49]. HR activity is crucial for repairing and re-starting the stalled replication forks to complete the telomere replication. Rad51C-depleted cells with or without telomerase both showed rapid telomere shortening phenotype likely as a result of HR deficiency [25]. In TAZ-deficient TNBC cells, we observed that telomeres were lost at the very early stage after TAZ depletion (48 h), and the activation of DNA damage responses at telomeres was also occurred (Figs. 2, 4). These TAZ deficiency-induced phenotypes in telomeres are quite similar to that of the Rad51C-deficient cells. Most importantly, recovery of Rad51C expression in TNBC cells indeed, at least partially, rescued the telomere shortening phenotype caused by TAZ deficiency (Fig. 5H–J). We also found TAZ-TEAD directly regulate the transcription of Rad51C by binding to its promoter (Fig. 6). Therefore, these results suggested that TAZ-maintained telomere integrity is likely achieved through regulating Rad51C and facilitating HR reactions.

As shown in our data, TAZ is overexpressed in TNBC (Additional file 1: Fig. S1 and Fig. 1A, B), and TNBC shows more CSC-like properties than non-TNBC [1]. In our study, we showed that knockdown of TAZ expression in TNBC leads to a significant decrease of telomere length and TAZ overexpression shows no obvious telomere length change in TNBC, non-TNBC cells and non-transformed mammary epithelial cells (Fig. 2). However, whether TAZ regulates telomere length in a TNBC-specific manner remains unclear. Other studies indicated the TAZ is involved in the regulation of cell senescence in non-TNBC cells, such as mammary tumor-derived cells, primary lung fibroblasts and normal human fibroblasts [27–29]. Considering the tightly relationship between cell senescence and telomere length, it’s still possible that TAZ also play a potential role in regulating telomere length in non-TNBC cells, other cancer type or even normal cells. However, more studies are needed in the future.

## Conclusions

In conclusion, our results revealed a novel role of TAZ in telomere regulation in TNBC cells. TAZ has been reported as an oncogene to promote cancer stem cell properties, proliferation, chemoresistance, and metastasis [2]. In this study, we showed that inhibition of TAZ might be a reasonable strategy to inhibit telomere maintenance in TNBC, and further leads to cancer cell senescence and a reduced cancer cell growth. Therefore, our data provided new evidence for the oncogenic effects of TAZ and supported the notion that TAZ may be a potential target for anti-cancer therapies in TNBC in the future.

## Supplementary Information

**Additional file 1.** Supplementary Materials.

## Data Availability

Not applicable.

## Abbreviations

ER: Estrogen receptor
PR: Progesterone receptor
TNBC: Triple negative breast cancer
TAZ: Transcriptional coactivator with PDZ-binding motif
TERRAs: Telomeric repeat-containing RNAs
HR: Homologous-recombination
hTERT: Human telomerase reverse transcriptase
TRF1: Telomeric repeat binding factor 1
TRF2: Telomeric repeat binding factor 2
POT1: Protection of telomeres 1
TIN2: TRF1-interacting protein 2
RAP1: Repressor/activator protein 1
ATM: Ataxia-telangiectasia mutated
ATR: Ataxia telangiectasia and Rad3 related
BRCA1: Breast cancer genes 1
CHK1: Checkpoint kinase 1
CHK2: Checkpoint kinase 2
53BP1: P53-binding protein 1
Rad51C: RAD51 Paralog C
TEAD4: TEA domain transcription factor 4
