# Supplementary material for: TAZ maintains telomere length in TNBC cells by mediating Rad51C expression
Source: Breast Cancer Res. 2021 Sep 6;23:89. doi: 10.1186/s13058-021-01466-z (PMC8422726; doi:10.1186/s13058-021-01466-z)
Supplement: Supplementary file 1 — Additional file 1. Supplementary Materials. [file 13058_2021_1466_MOESM1_ESM.pdf]

Supplementary Information

Supplementary Figure Legends

Figure S1. TAZ is highly expressed in TNBC. A, mRNA expression levels of TAZ in TNBC and non-TNBC were analyzed with Breast Cancer Integrative Platform database (www.omicsnet.org/bcancer/database). **B,** mRNA expression levels of TAZ in different breast cancer cell lines are examined with Cancer Cell Line Encyclopedia database (https://portals.broadinstitute.org/ccle). The red columns represent the TNBC cells, the blue ones represent the $HER2^+$ Breast Cancer cells, the green ones represent the luminal breast cancer. **C,** Copy number variation (CNV) analysis was performed by using the Breast Cancer Integrative Platform database (http://www.omicsnet.org/bcancer/database).

Figure S2. Early passages of TAZ-depleted cells show shortened telomeres but no changes of hTERT expression. A and B, Western blot analyses of TAZ in the indicated MDA-MB-231 and BT549 cells. Quantification of Western blots is shown in Fig. S5 O and P. **C and D,** qPCR analyses to determine hTERT mRNA levels in the indicated MDA-MB-231 and BT549 cells. **E and F,** Western blot analyses of hTERT in the indicated MDA-MB-231 and BT549 cells. Quantification of MDA-MB-231 Western blots is shown in Fig. S5Q. **G and H,** Telomere-specific qPCR analyses in the indicated MDA-MB-231 and BT549 cells to determine the relative telomere length. **I and J,** Southern analysis of TRFs in the indicated MDA-MB-231 and BT549 cells to determine the relative telomere length. For A-J, cells were lyzed after 48 h of transfection, representing the short-term knockdown of TAZ expression. **K,** TAZ-depleted MDA-MB-231 cells were prepared with shRNA and cultured for different passages as indicated. Telomere-specific qPCR analyses were performed. A progressively shortening of telomeres during passage elongation in TAZ-depleted cells was observed. **L,** The mRNA levels of TAZ and hTERT were examined by qPCR in BT549 cells transfected with TAZ-specific shRNA for 48 h. Data are presented as mean±SEM. At least three repeats were carried out for each test. The p values were determined by one-way ANOVA followed by Tukey's multiple-comparisons. NS, not significant, *$p<0.05$, **$p<0.01$, ***$p<0.001$.

Figure S3. Early passages of TAZ-depleted TNBC cells show no changes of shelterin proteins. Western blot analysis of shelterin proteins in short-term TAZ knockdown (transfected with siRNA for 48 h) MDA-MB-231 cells (left panel) and BT549 cells (right panel). Quantification of MDA-MB-231 Western blots is shown in Fig. S5R.

Figure S4. Knockdown of TAZ activates TERRAs transcription and p53 nuclear translocation. A-D, qPCR analyses showing the changes of the expression of distinct TERRA transcripts from different chromosomes (chr-

2q, chr-9p, chr-10q, chr-13q, chr-17p and chr-xqyq) in MDA-MB-231 and BT549 cells with short-term TAZ knockdown (transfected with siRNA for 48 h) or long-term TAZ knockdown (infected with shRNA and cultured >6 passages). Data are presented as mean±SEM. At least three repeats were carried out for each test. The p values were determined by one-way ANOVA followed by Tukey's multiple-comparisons. NS, not significant, *p<0.05, **p<0.01, ***p<0.001.

Figure S5. Quantification of western blotting. A, Quantification of the data obtained in Fig1A. **B,** Quantification of the data obtained in Fig1C. **C,** Quantification of the data obtained in Fig2A. **D,** Quantification of the data obtained in Fig2D. **E,** Quantification of the data obtained in Fig2G. **F,** Quantification of the data obtained in Fig3C. **G,** Quantification of the data obtained in Fig4A. **H,** Quantification of the data obtained in Fig4B. **I,** Quantification of the data obtained in Fig4D. **J,** Quantification of the data obtained in Fig5A. **K,** Quantification of the data obtained in Fig5C. **L,** Quantification of the data obtained in Fig5F. **M,** Quantification of the data obtained in Fig5H. **N,** Quantification of the data obtained in Fig6B. **O,** Quantification of the data obtained in FigS2A. **P,** Quantification of the data obtained in FigS2B. **Q,** Quantification of the data obtained in FigS2E. **R,** Quantification of the data obtained in FigS3. Data are presented as mean±SEM. At least three repeats were carried out for each test. Statistical analyses were performed with unpaired Student's t-test between two groups and one -way ANOVA followed by Tukey's multiple-comparisons for multiple groups. NS, not significant, *p<0.05, **p<0.01, ***p<0.001.

Supplementary Tables

Supplementary Table S1

**si-Cont.**	**UUCUCCGAACGUGUCACGUTT**
**si-TAZ#1**	**GCUCAUGAGUAUGCCCAAUTT**
**si-TAZ#2**	**GGUACUUCCUCAAUCACAUTT**
**sh-Cont.**	**TTCTCCGAACGTGTCACG**
**sh-TAZ#1**	**GATGAATCAGCCTCTGAAT**
**sh-TAZ#2**	**CCTTTCTAACCTGGCTGTA**
**si-CTNNB1#1**	**GGAUGUGGAUACCUCCCAATT**
**si-CTNNB1#2**	**CCCACUAAUGUCCAGCGUUTT**
**si-p53**	**CCACUGGAUGGAGAAUAUUTT**
**si-RAD51C#1**	**CCAAAGAAGGAGCUAAUAATT**
**si-RAD51C#2**	**CCUUGCUACUGCCUGCAUUTT**

Supplementary Table S2

TAZ-F	GCUCAUGAGUAUGCCCAAUTT
TAZ-R	GGUACUUCCUCAAUCACAUTT
IL-1a-F	GATCAGTACCTCACGGCTGC
IL-1a-R	GCATCTCCTTCAGCAGCACT
IL-1b-F	CCAGCTACGAATCTCCGACC
IL-1b-R	GGGAAAGAAGGTGCTCAGGT
IL6-F	CCAGAGCTGTGCAGATGAGT
IL6-R	GCATTTGTGGTTGGGTCAGG
IL8-F	CTCCAAACCTTTCCACCCCA
IL8-R	TTCTCCACAACCCTCTGCAC
Tel-F	GGTTTTTGAGGGTGAGGGTGAGGGTGAGGGTGAGGGT
Tel-R	TCCCGACTATCCCTATCCCTATCCCTATCCCTATCCCTA
HBG-F	GCTTCTGACACAACTGTGTTCACTAGC
HBG-R	CACCAACTTCATCCACGTTCACC
hTERT-F	GCCTTCAAGAGCCACGTC
hTERT-R	CCACGAACTGTCGCATGT
Terc-F	GTGGTGGCCATTTTTTGTCTAAC
Terc-R	TGCTCTAGAATGAACGGTGGAA
Rad51C-F	GAAAAACACAAGGGAGAGGAAC
Rad51C-R	GTAACTCTGTGTAGTCACGACA
TERRA-chr-2q-F	GCCTTGCCTTGGGAGAATCT
TERRA-chr-2q-R	AAAGCGGGAAACGAAAAGC
TERRA-chr-9p-F	GAGATTCTCCCAAGGCAAGG
TERRA-chr-9p-R	ACATGAGGAATGTGGGTGTTAT
TERRA-chr-10q-F	AAAGCGGGAAACGAAAAGC
TERRA-chr-10q-R	GCCTTGCCTTGGGAGAATCT
TERRA-chr-13q-F	CCTGCGCACCGAGATTCT
TERRA-chr-13q-R	GCACTTGAACCCTGCAATACAG

**TERRA-chr-17p-F**	**CTTATCCACTTCTGTCCCAAGG**
**TERRA-chr-17p-R**	**CCCAAAGTACACAAAGCAATCC**
**TERRA-chr-XqYq-F**	**CCCCTTGCCTTGGGAGAA**
**TERRA-chr-XqYq-R**	**GAAAGCAAAAGCCCCTCTGA**
**GAPDH-F**	**AGAAGGCTGGGGCTCATTTG**
**GAPDH-R**	**AGGGGCCATCCACAGTCTTC**

Figure S1

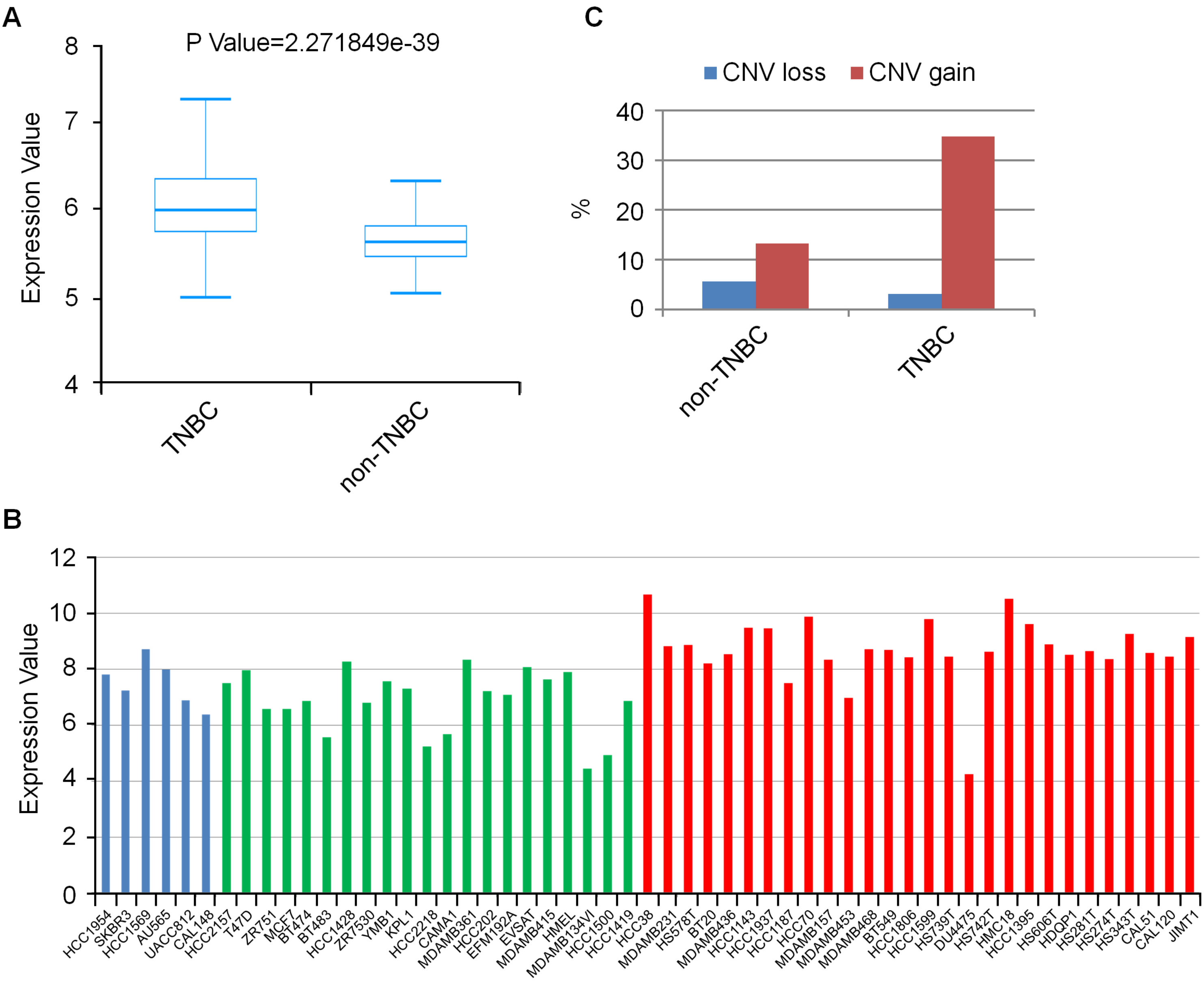
A
P Value=2.271849e-39
Expression Value
TNBC
non-TNBC
C
CNV loss
CNV gain
%
non-TNBC
TNBC
B
Expression Value
HCC1954
SKBR3
HCC1569
AU565
UACC812
CAL148
HCC2157
T47D
ZR751
MCF7
BT474
BT483
HCC1428
ZR7530
YMB1
KPL1
HCC2218
CAMA1
MDAMB361
HCC202
EFM192A
EVSAT
MDAMB415
HMEL
MDAMB134VI
HCC1500
HCC1419
HCC38
MDAMB231
HS578T
BT20
MDAMB436
HCC1143
HCC1937
HCC1187
HCC70
MDAMB157
MDAMB453
MDAMB468
BT549
HCC1806
HCC1599
HS739T
DU4475
HS742T
HMC18
HCC1395
HS606T
HDQP1
HS281T
HS274T
HS343T
CAL51
CAL120
JIMT1

Figure S2

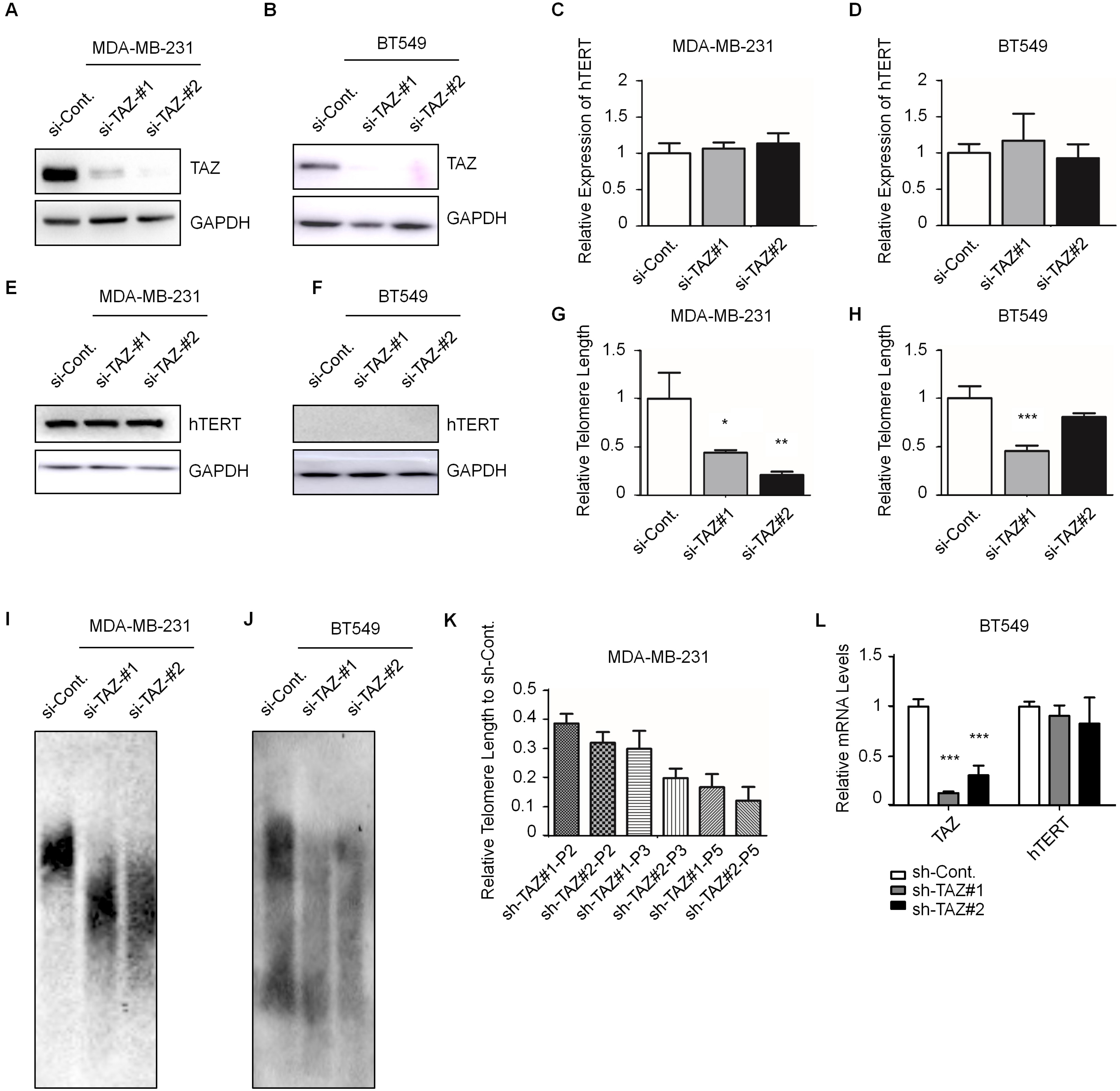

A
MDA-MB-231
si-Cont.
si-TAZ-#1
si-TAZ-#2
TAZ
GAPDH
B
BT549
si-Cont.
si-TAZ-#1
si-TAZ-#2
TAZ
GAPDH
C
MDA-MB-231
Relative Expression of hTERT
si-Cont.
si-TAZ#1
si-TAZ#2
D
BT549
Relative Expression of hTERT
si-Cont.
si-TAZ#1
si-TAZ#2
E
MDA-MB-231
si-Cont.
si-TAZ-#1
si-TAZ-#2
hTERT
GAPDH
F
BT549
si-Cont.
si-TAZ-#1
si-TAZ-#2
hTERT
GAPDH
G
MDA-MB-231
Relative Telomere Length
si-Cont.
si-TAZ#1
si-TAZ#2
H
BT549
Relative Telomere Length
si-Cont.
si-TAZ#1
si-TAZ#2
I
MDA-MB-231
si-Cont.
si-TAZ-#1
si-TAZ-#2
J
BT549
si-Cont.
si-TAZ-#1
si-TAZ-#2
K
MDA-MB-231
Relative Telomere Length to sh-Cont.
sh-TAZ#1-P2
sh-TAZ#2-P2
sh-TAZ#1-P3
sh-TAZ#2-P3
sh-TAZ#1-P5
sh-TAZ#2-P5
L
BT549
Relative mRNA Levels
TAZ
hTERT
sh-Cont.
sh-TAZ#1
sh-TAZ#2

Figure S3

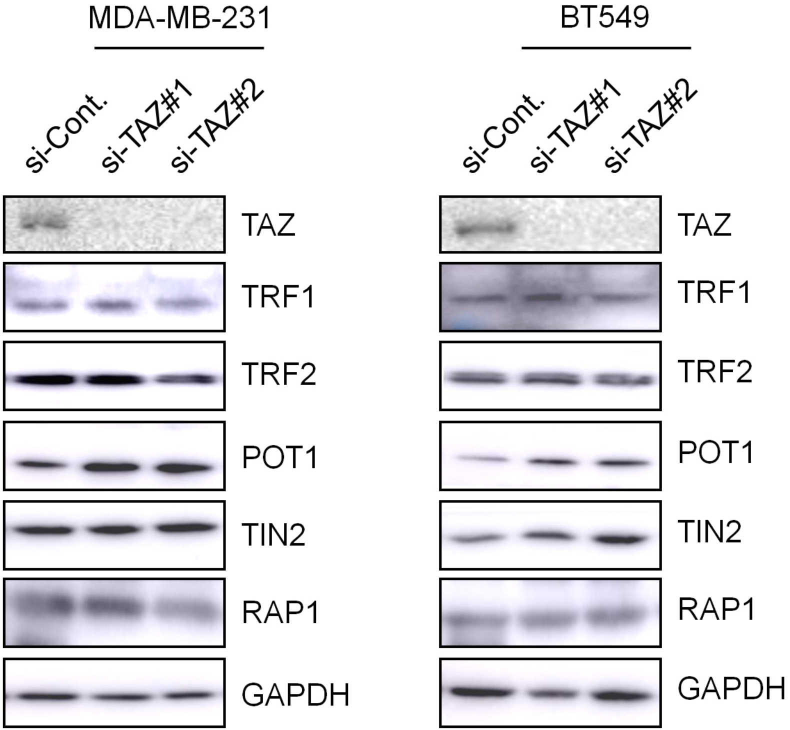
MDA-MB-231
BT549
si-Cont.
si-TAZ#1
si-TAZ#2
si-Cont.
si-TAZ#1
si-TAZ#2
TAZ
TRF1
TRF2
POT1
TIN2
RAP1
GAPDH
TAZ
TRF1
TRF2
POT1
TIN2
RAP1
GAPDH

Figure S4

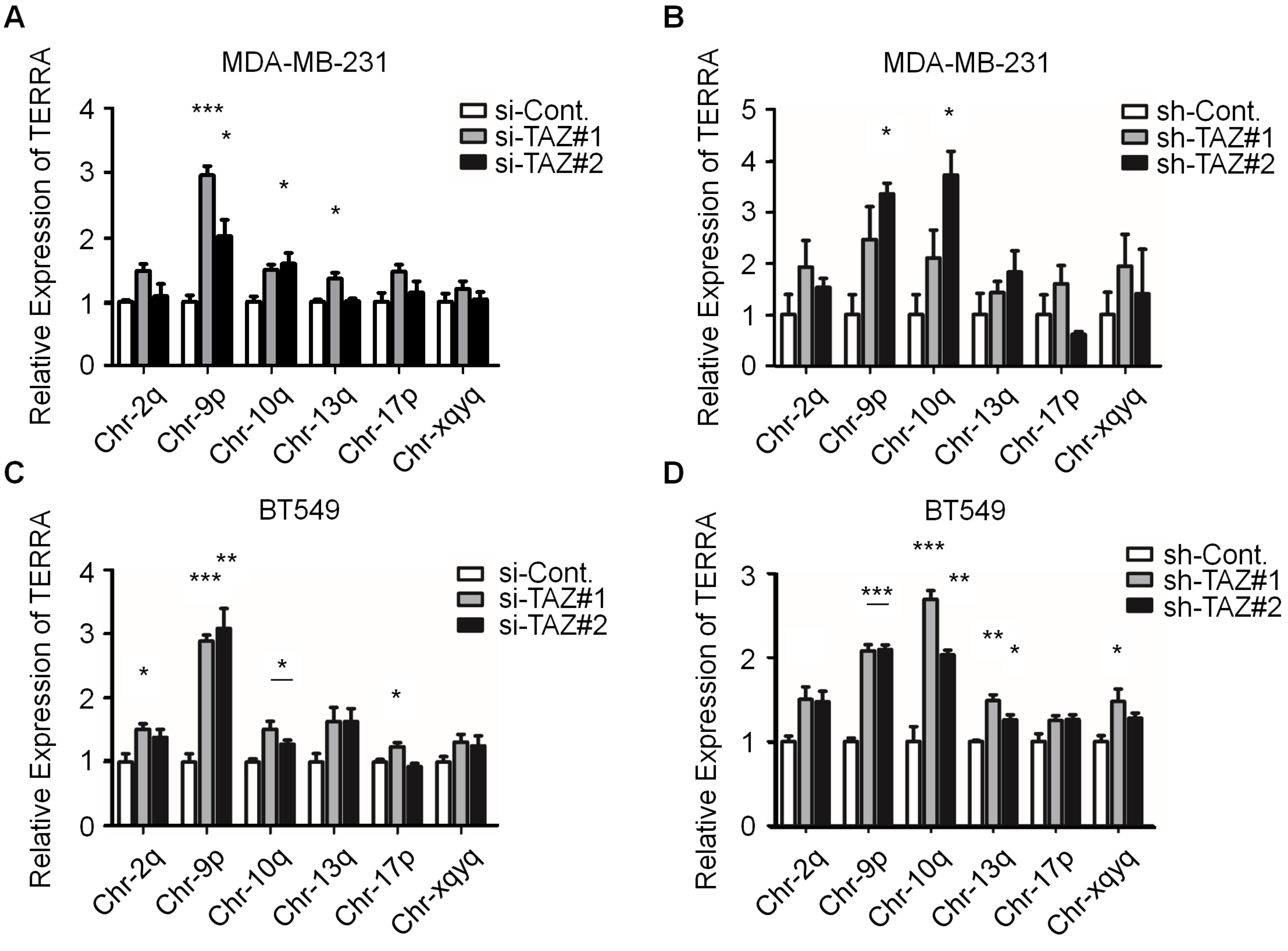

A
MDA-MB-231
Relative Expression of TERRA
si-Cont.
si-TAZ#1
si-TAZ#2
Chr-2q
Chr-9p
Chr-10q
Chr-13q
Chr-17p
Chr-xqyq
B
MDA-MB-231
sh-Cont.
sh-TAZ#1
sh-TAZ#2
C
BT549
D
BT549

Figure S5

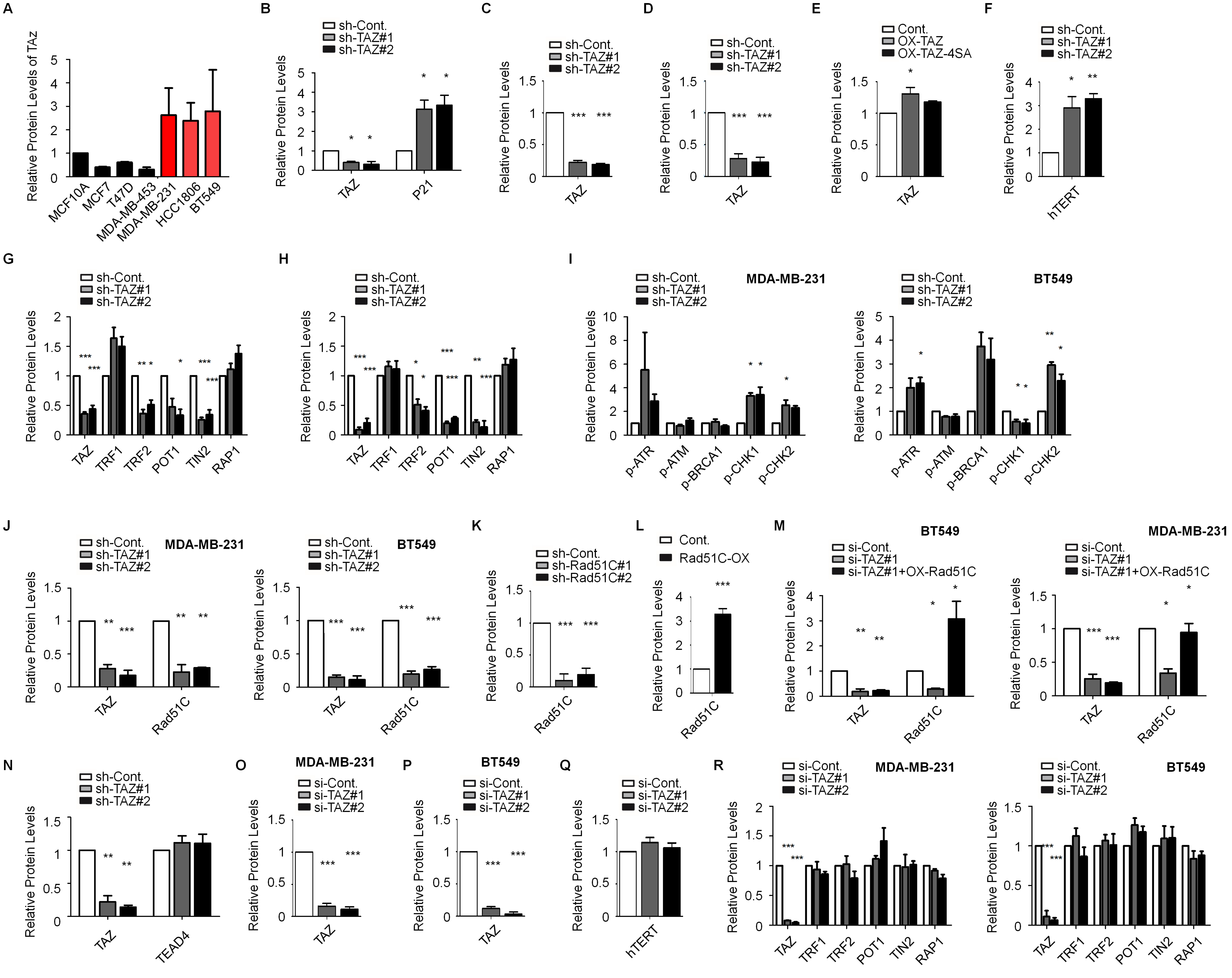
A
Relative Protein Levels of TAz
MCF10A
MCF7
T47D
MDA-MB-453
MDA-MB-231
HCC1806
BT549
B
sh-Cont.
sh-TAZ#1
sh-TAZ#2
Relative Protein Levels
TAZ
P21
C
sh-Cont.
sh-TAZ#1
sh-TAZ#2
Relative Protein Levels
TAZ
D
sh-Cont.
sh-TAZ#1
sh-TAZ#2
Relative Protein Levels
TAZ
E
Cont.
OX-TAZ
OX-TAZ-4SA
Relative Protein Levels
TAZ
F
sh-Cont.
sh-TAZ#1
sh-TAZ#2
Relative Protein Levels
hTERT
G
sh-Cont.
sh-TAZ#1
sh-TAZ#2
Relative Protein Levels
TAZ
TRF1
TRF2
POT1
TIN2
RAP1
H
sh-Cont.
sh-TAZ#1
sh-TAZ#2
Relative Protein Levels
TAZ
TRF1
TRF2
POT1
TIN2
RAP1
I
sh-Cont.
sh-TAZ#1
sh-TAZ#2
MDA-MB-231
Relative Protein Levels
p-ATR
p-ATM
p-BRCA1
p-CHK1
p-CHK2
sh-Cont.
sh-TAZ#1
sh-TAZ#2
BT549
Relative Protein Levels
p-ATR
p-ATM
p-BRCA1
p-CHK1
p-CHK2
J
sh-Cont.
sh-TAZ#1
sh-TAZ#2
MDA-MB-231
Relative Protein Levels
TAZ
Rad51C
sh-Cont.
sh-TAZ#1
sh-TAZ#2
BT549
Relative Protein Levels
TAZ
Rad51C
K
sh-Cont.
sh-Rad51C#1
sh-Rad51C#2
Relative Protein Levels
Rad51C
L
Cont.
Rad51C-OX
Relative Protein Levels
Rad51C
M
BT549
si-Cont.
si-TAZ#1
si-TAZ#1+OX-Rad51C
Relative Protein Levels
TAZ
Rad51C
MDA-MB-231
si-Cont.
si-TAZ#1
si-TAZ#1+OX-Rad51C
Relative Protein Levels
TAZ
Rad51C
N
sh-Cont.
sh-TAZ#1
sh-TAZ#2
Relative Protein Levels
TAZ
TEAD4
O
MDA-MB-231
si-Cont.
si-TAZ#1
si-TAZ#2
Relative Protein Levels
TAZ
P
BT549
si-Cont.
si-TAZ#1
si-TAZ#2
Relative Protein Levels
TAZ
Q
si-Cont.
si-TAZ#1
si-TAZ#2
Relative Protein Levels
hTERT
R
si-Cont.
si-TAZ#1
si-TAZ#2
MDA-MB-231
Relative Protein Levels
TAZ
TRF1
TRF2
POT1
TIN2
RAP1
si-Cont.
si-TAZ#1
si-TAZ#2
BT549
Relative Protein Levels
TAZ
TRF1
TRF2
POT1
TIN2
RAP1
